# EGR2-mediated regulation of m^6^A reader IGF2BP proteins drive RCC tumorigenesis and metastasis via enhancing S1PR3 mRNA stabilization

**DOI:** 10.1038/s41419-021-04038-3

**Published:** 2021-07-29

**Authors:** Yufan Ying, Xueyou Ma, Jiajie Fang, Shiming Chen, Weiyu Wang, Jiangfeng Li, Haiyun Xie, Jian Wu, Bo Xie, Ben Liu, Xiao Wang, Xiangyi Zheng, Liping Xie

**Affiliations:** 1 Department of Urology, First Affiliated Hospital, School of Medicine, Zhejiang University, 310000 Hangzhou, Zhejiang China; 2 Cancer Center, Zhejiang University, 310058 Hangzhou, Zhejiang China

**Keywords:** Cancer, Kidney diseases

## Abstract

Emerging discoveries of dynamic and reversible N6-methyladenosine (m^6^A) modification on RNA in mammals have revealed the key roles of the modification in human tumorigenesis. As known m^6^A readers, insulin-like growth factor 2 mRNA-binding proteins (IGF2BPs) are upregulated in most cancers and mediates the enhancement of m^6^A-modified mRNAs stability. However, the mechanisms of IGF2BPs in renal cell cancer (RCC) still remain unclear. Bioinformatic analysis and RT-qPCR were performed to evaluate the expression of IGF2BPs and m^6^A writer Wilms tumor 1-associating protein (WTAP) in RCC samples and its correlation with patient prognosis. In vitro, in vivo biological assays were performed to investigate the functions of IGF2BPs and WTAP in RCC. Chromatin immunoprecipitation-qPCR (ChIP-qPCR) combined with bioinformatics analysis and following western blot assay, dual-luciferase reporter assays were performed to validate the regulatory relationships between transcription factor (TF) early growth response 2 (EGR2) and potential target genes IGF2BPs. RNA sequencing (RNA-seq), methylated RNA immunoprecipitation-qPCR (MERIP-qPCR), RIP-qPCR, m^6^A dot blot, and dual-luciferase reporter assays combined with bioinformatics analysis were employed to screen and validate the direct targets of IGF2BPs and WTAP. Here, we showed that early growth response 2 (EGR2) transcription factor could increase IGF2BPs expression in RCC. IGF2BPs in turn regulated sphingosine-1-phosphate receptor 3 (S1PR3) expression in an m^6^A-dependent manner by enhancing the stability of S1PR3 mRNA. They also promoted kidney tumorigenesis via PI3K/AKT pathway. Furthermore, IGF2BPs and WTAP upregulation predicted poor overall survival in RCC. Our studies showed that the EGR2/IGF2BPs regulatory axis and m^6^A-dependent regulation of S1PR3-driven RCC tumorigenesis, which enrich the m^6^A-modulated regulatory network in renal cell cancer. Together, our findings provide new evidence for the role of N6-methyladenosine modification in RCC.

## Introduction

In 2018, 400,000 new diagnoses and over 170,000 deaths due to renal cancer were reported [1]. Renal cell carcinoma (RCC) comprises about 90% of all renal-originated tumors [2], with 35% of RCC patients develop metastases [3]. About 25–30% of patients with local metastases who receive radical nephrectomy develop metastases within 5 years [4]. Thus, new therapeutic approaches should be developed.

The N6-methyladenosine modification was first discovered in 1974 [5]. Since then, the mechanisms of RNA modification have remained elusive, until the first RNA demethylase, the fat mass, and obesity-associated protein (FTO), revealed that m^6^A is reversible in 2011 [6]. Using the methylated RNA immunoprecipitation-sequencing method, m^6^A modifications were found to be enriched in the 3’-untranslated regions (3’UTRs) and 5’-untranslated regions (5’UTRs) [7], which regulate RNA alternative splicing, mRNA degradation, and translation [8].

The m^6^A modification is determined by the methyltransferase complex (MTC), which contains methyltransferases called “writers” [9]. The Wilms tumor 1-associating protein (WTAP) stabilizes MTC localization and substrate recruitment [10–12]. The RNA containing m^6^A modification selectively binds to divergent m^6^A readers, leading to diverse modifications. Recent studies show that IGF2BPs, an additional family of readers, stabilize the translation of target mRNA, in contrast to YTHDF2 [13, 14].

The insulin-like growth factor 2 mRNA-binding protein 1-3 (IGF2BP1-3) is primarily expressed in various cancers [15]. The IGF2BP1-3 family was initially found to regulate fetal growth factor (IGF2) and RNA metabolism [16]. Since IGF2BPs are m^6^A-binding proteins, IGF2BPs promote tumor initiation and metastasis by stabilizing the m^6^A-containing mRNAs [17–21]. So far, few studies have explored the role of IGF2BPs in renal cancer.

## Methods

### Cell culture and transfection

786-O, CAKI-1 cells were purchased from the Cell Bank of the Chinese Academy of Sciences (Shanghai, China). Cell culture experiments were conducted according to the institutional guidelines. Cell transfection assays were conducted using Polyplus transfection reagent (Proteintech, Polyplus Transfection Strasbourg, France). siRNAs used are shown in Supplementary Table S1.

### Clinical tissue sample

Twenty-four paired kidney cancer tissues and adjacent non-cancerous tissues were obtained from patients with newly diagnosed renal cancer. The clinical specimens were collected at the First Affiliated Hospital of Medical College, Zhejiang University after informed consent and the approval of the Ethics Committee of Zhejiang University. Clinicopathological characteristics of the patients are presented in Table 1.

**Table 1:** Clinical data of the RCC patients (*n* = 24).

No.	Age	Sex	Pathologic diagnosis	pT stage	Fuhrman grade
1	55	Male	Clear cell	T1b	II
2	55	Male	Clear cell	T2a	II
3	36	Male	Papillary	T1a	I
4	49	Female	Clear cell	T2a	II
5	55	Male	Clear cell	T3a	II
6	59	Female	Clear cell	T1b	I
7	68	Female	Clear cell	T3a	II
8	74	Male	Clear cell	T1b	I
9	62	Male	Clear cell	T1b	II
10	75	Male	Clear cell	T1a	I
11	43	Male	Clear cell	T1a	I
12	59	Male	Clear cell	T3b	III
13	58	Female	Clear cell	T1b	I
14	54	Female	Clear cell	T3a	I
15	60	Male	Clear cell	T4	II
16	63	Female	Clear cell	T1b	II
17	65	Female	Clear cell	T1b	I
18	54	Male	Clear cell	T3a	II
19	57	Male	Clear cell	T1a	I
20	56	Male	Clear cell	T2a	III
21	53	Female	Clear cell	T1b	I
22	67	Male	Clear cell	T2a	I
23	51	Female	Clear cell	T1b	II
24	65	Male	Clear cell	T1b	I

### RNA-seq

Total RNA was isolated from the 786-O cells using Trizol following the manufacturer’s protocol (Invitrogen). The library construction and RNA sequencing were performed and analyzed by Guangzhou RiboBio Co., Ltd (Guangzhou, China) using Illumina HiSeq xten as previously reported [22]. Differentially expressed genes were used to construct a heatmap and KEGG ontology enrichment analyses. For KEGG enrichment analysis, a *P* value < 0.05 was used as the threshold to determine significantly enriched gene sets.

### MeRIP-quantitative real-time PCR

The MeRIP-quantitative real-time PCR was performed as per Magna methylated RNA immunoprecipitation kit guidelines (MeRIP) m^6^A Kit (Merck Millipore). Briefly, total RNA was isolated from 786-O and CAKI-1 cell lines fragmented to 100 nt and subjected to immunoprecipitation with m^6^A (Abcam, ab208577) or immunoglobulin G (IgG) antibody-conjugated beads for 2 h at 4 °C. Bound RNA was eluted and purified with the RNA purification kit (QIAGEN). The purified RNA was analyzed by RT-qPCR. The primers used are listed in Supplementary Table S1.

### RIP-quantitative real-time PCR

RIP experiments were performed using the Magna RIP kit (Merck Millipore). Primarily, 786-O and CAKI-1 cells were cultured in several 15-cm plates. The lysates were centrifuged and the supernatant was incubated with magnetic beads loaded with 15 μg of antibody (IGF2BP1, IGF2BP2, IGF2BP3, WTAP, Proteintech) or IgG overnight at 4 °C. Then the magnetic-bead-bound complex was incubated with proteinase K, and bound RNA was isolated and purified for the following RT-qPCR assay.

### RNA m^6^A dot blot assay

The total RNA extracted from renal cancer cells or tissues was adjusted to 50 ng/μL with 36 μL RNase-free water. After removal of RNA secondary structure and treatment with ice-cold 20× saline sodium citrate (SSC) solution (Sigma-Aldrich), RNA samples (100 or 200 ng) were loaded onto N+ membrane (GE health) in dot blot apparatus (Bio-Rad). It was cross-linked by ultraviolet radiation and stained by methylene blue (Sigma-Aldrich) then incubated with m^6^A antibody. Results were analyzed by ImageJ software.

### ChIP-quantitative real-time PCR

Chromatin immunoprecipitation (ChIP) experiments were performed as described previously [23]. In brief, 1 × 10^7^ 786-O and CAKI-1 cells were treated with formaldehyde, quenched, and harvested in lysis buffer. For ChIP assay, total sheared chromatin was incubated with control (anti-IgG, Millipore) or anti-EGR2 (sc-518117, Santa Cruz) antibodies overnight. After washing, chromatin was eluted in the elution buffer, treated with Proteinase K, and the cross-links were reversed. DNA was finally eluted as per the manufacturer’s protocol and analyzed by qPCR. Primers are summarized in Supplementary Table S1.

### Lentiviruses transfection

Lentiviruses, Cas9, and luciferase vector, used for in vivo imaging, were obtained from Genechem (Shanghai, China). The Cas9 Vector, GV392 vector, (U6-sgRNA-EF1a-Cas9-FLAG-P2A-puromycin) contained IGF2BP1, IGF2BP2, IGF2BP3, and WTAP sgRNAs. The luciferase vector, GV542 vector, contained Ubi-MCS-firefly_Luciferase-SV40-neomycin. Lentivirus infection was conducted following the manufacturer’s instructions. The sgRNA sequences are listed in Supplementary Table S1.

### Western blot assay

Western blot assay was conducted as previously described [24]. Antibodies used in this study were as follows: anti-GAPDH (10494-1-AP, Proteintech), anti-AKT (C67E7, Cell Signaling Technology), anti-pAKT (S473) (193H12, Cell Signaling Technology), anti-PI3K (Y388, Abcam), anti-PI3K (p110) (Y384, Abcam), anti-S1PR3 (EPR454, Abcam), anti-EGR2 (EPR4004, Abcam), anti-IGF2BP1 (22803-1-AP, Proteintech), anti-IGF2BP2 (11601-1-AP, Proteintech), anti-IGF2BP3 (14642-1-AP, Proteintech), anti-WTAP (10200-1-AP, Proteintech).

### RNA isolation and quantitative real-time PCR

Total RNA was extracted from cell lines and tissues by RNAiso plus (Takara, Japan) and analyzed by RT-qPCR as described [24]. All the primers used are shown in Supplementary Table S1.

### Colony formation and cell migration assay

In total, 500 of transfected cells or stable knockdown cell lines were seeded in 6-well plates per well and cultured for 2 weeks. Cells were fixed using methanol and 0.2% crystal violet. Next, colony formation was calculated as previously described [25].

Cell migration was evaluated using transwell chambers (Merck Millipore). In total, 4 × 10^4^ 786-O cells and 8 × 10^4^ CAKI-1 cells were suspended in 0.2 mL serum-free medium and added to chambers. After incubation for 24 h, the migrated cells were fixed on the surface of the chamber using methanol and 0.2% crystal violet. And migration abilities were evaluated as previously described [25].

### Dual-luciferase reporter assay

Plasmids containing potential target region (wild-type) or mutant target region (mutated-type) were designed by Sangon, China. A dual-luciferase reporter assay was conducted as previously described [25]. The 786-O cells were seeded in 96-well plates and co-transfected with 12.5 nmol/L siRNA or NC and 25 ng of the above-constructed target reporter. The relative luciferase activity was measured by the dual-luciferase reporter assay (Promega, Madison, USA).

### Animal experiments

Subcutaneous and orthotopic transplantation models were prepared as described previously [25, 26]. All animal protocols followed institutional guidelines of the First Affiliated Hospital, School of Medicine, Zhejiang University.

### Statistical analysis

Data are presented as mean ± SD and were prepared using GraphPad Prism 8.0 (La Jolla, CA, USA). Overall survival rate was calculated by the Kaplan–Meier analysis and log-rank test. Groups were compared by a two‐tailed Student’s *t* test. Statistical significance was defined as *P* value of <0.05.

## Results

### High WTAP and IGF2BPs expression correlate with overall survival of patients with RCC

**Fig. 1:**
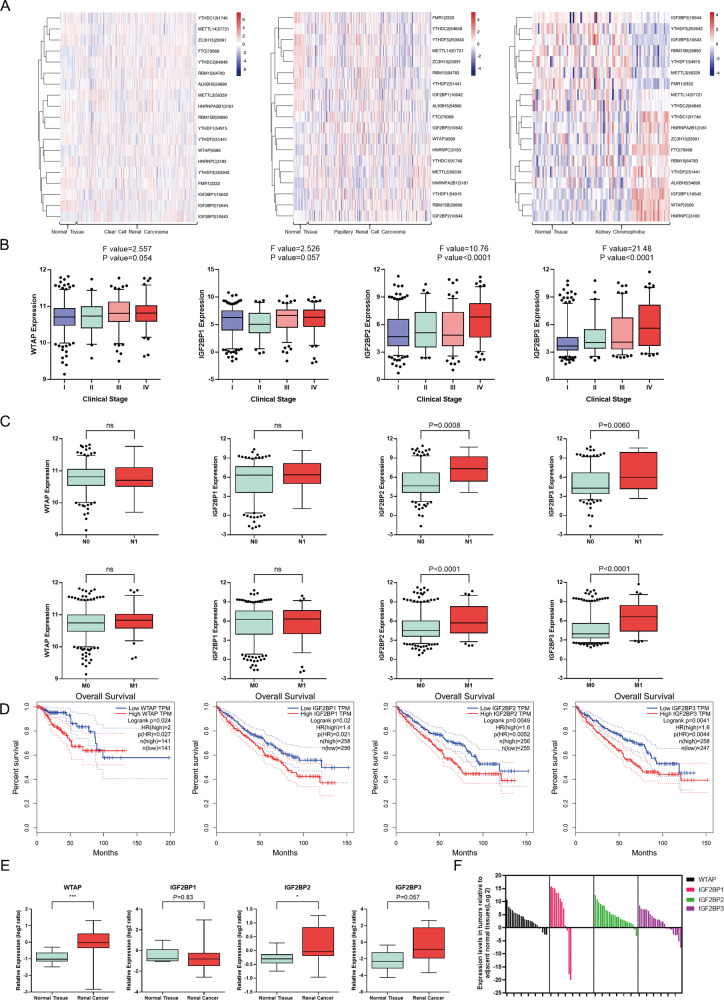
Deregulation of m^6^A methylation enzymes leads to poor prognosis of RCC patients. **A** TCGA data showed the deregulated m^6^A regulators in different RCC patients. **B**, **C** Clinical features of RCC patients associated with WTAP, IGF2BPs genes expression. **D** Elevated expression of WTAP and IGF2BPs correlate with poor prognosis in RCC patients. **E** The mRNA levels of WTAP and IGF2BPs in RCC tissues and normal tissues according to the Oncomine database. **F** The mRNA levels of WTAP and IGF2BPs in RCC tissues relative to adjacent nonmalignant tissues are shown. Values in **B**, **C**, and **E** are mean ± SEM. *P < 0.05; **P < 0.01; ***P < 0.001.

To determine the role of m^6^A RNA modification in RCC, a heatmap showing expression patterns of m^6^A regulators across various kidney tissues in the TCGA database was designed (Fig.  1A). The key m^6^A writer WTAP was significantly upregulated in RCC tissues. Moreover, high mRNA levels of IGF2BP2 and IGF2BP3 were associated with late clinical stage, nodal involvement, and distant metastasis. Although not statistically significant, a similar trend was observed when analyzing WTAP and IGF2BP1 (Fig. 1B, C). RCC patients with high WTAP mRNA levels, as well as high IGF2BPs levels, had a shorter survival time of than those with downregulated expression (Fig. 1D). To further confirm the role of WTAP and IGF2BPs in RCC, mRNA levels were measured by RT-qPCR assay in 24 pairs of clinically matched adjacent noncancerous kidney tissues and human renal cell carcinoma tissues. Similarly, WTAP and IGF2BPs were higher in kidney cancer tissues than in adjacent normal controls (Fig.1E). This indicates that upregulation of WTAP and IGF2BPs correlates with poor prognosis in RCC patients.

### EGR2 activates transcription of IGF2BPs in RCC

**Fig. 2:**
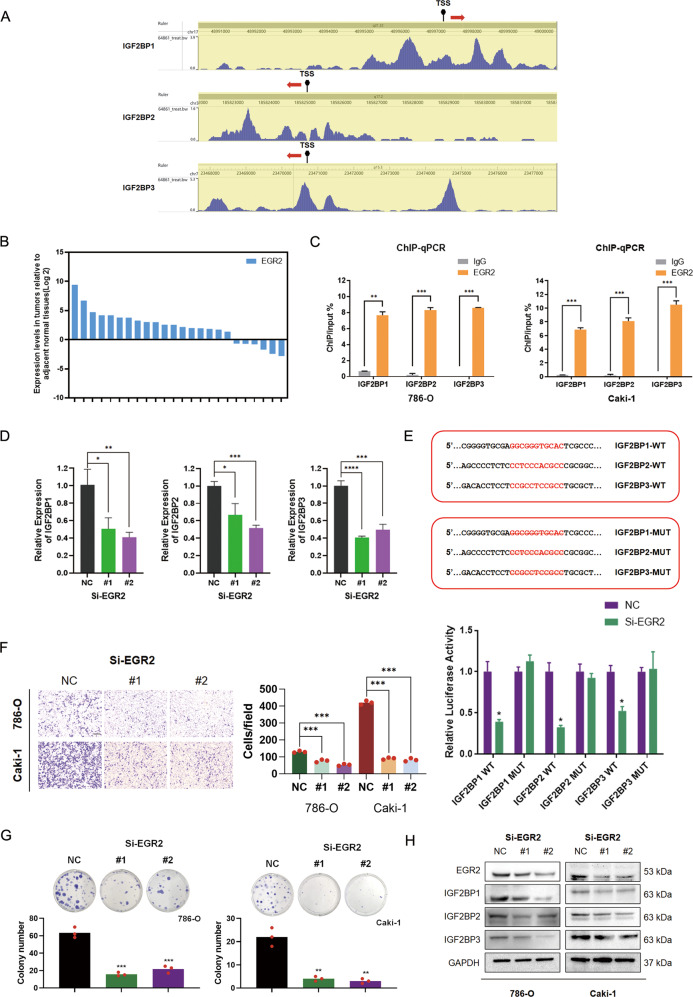
EGR2 contributes to the deregulation of IGF2BPs in RCC patients and promotes RCC migration and proliferation. **A** Enriched peaks were identified using the ChIP-seq data. **B** EGR2 were elevated in 18 (75%) RCC tissues relative to adjacent nonmalignant tissues. **C** RT-qPCR analyses for ChIP using EGR2 antibodies were performed. **D** The mRNA levels of IGF2BP1-3 were markedly decreased with EGR2 knockdown. **E** Reporter gene analysis using the luciferase reporter construct individually driven by the IGF2BPs promoters (shown schematically at the bottom). **F**, **G** Knockdown of EGR2 suppressed migration and proliferation viabilities in RCC cells. Scale bar 250 μm. **H** Western blot assays were performed to identify the protein levels of IGF2BP genes when EGR2 was knockdown. Values in **C**, **D**, and **E** are mean ± SEM. *P < 0.05; **P < 0.01; ***P < 0.001.

To explore how high IGF2BPs expression affects RCC patients in the late clinical stage, transcription factor binding site analysis for IGF2BPs promoter was conducted using the Cistrome Data Browser database (http://cistrome.org/db/#/). Results indicated that the IGF2BPs promoter region contained the putative binding sites of transcription factor EGR2 (Fig. 2A). Elevated EGR2 expressions were observed in most RCCs tissues compared with normal tissues (Fig. 2B). Furthermore, gene expression analysis using the data obtained from the LinkedOmics database revealed a subset of genes positively correlated with IGF2BPs [27]. A weak positive correlation was found between gene EGR2 and IGF2BPs expression (Supplementary Fig. 3F). We, therefore, performed a chromatin immunoprecipitation assay (ChIP) to determine whether EGR2 directly binds to IGF2BPs. We identified EGR2 binding sites using the JASPAR programs and analyzed the sequence of IGF2BPs promoter and designed primers for RT-qPCR for amplifying a region containing the potential binding sites [28]. Notably, ChIP assays demonstrated that EGR2 directly bound to the promoter of IGF2BPs (Fig. 2C), and knockdown of EGR2 markedly decreased the mRNA levels of IGF2BPs (Fig. 2D). As a tool to determine the functional activity of EGR2, we constructed a dual-luciferase reporter assay. Knockdown of EGR2 also markedly decreased the luciferase activity of the wild-type reporters (Fig. 2E). In further experiments, EGR2 gene was knocked down by siRNA transfection, and results were confirmed by western blotting (Fig. 2H). EGR2 silencing downregulated IGF2BPs expression (Fig. 2H). In addition, EGR2 expression positively regulated the migration and proliferation of RCC cells (Fig. 2F, G). These results suggested that EGR2 acts as an oncogene via directly binding to genes such as IGF2BPs.

### WTAP and IGF2BPs promote RCC migration and proliferation

**Fig. 3:**
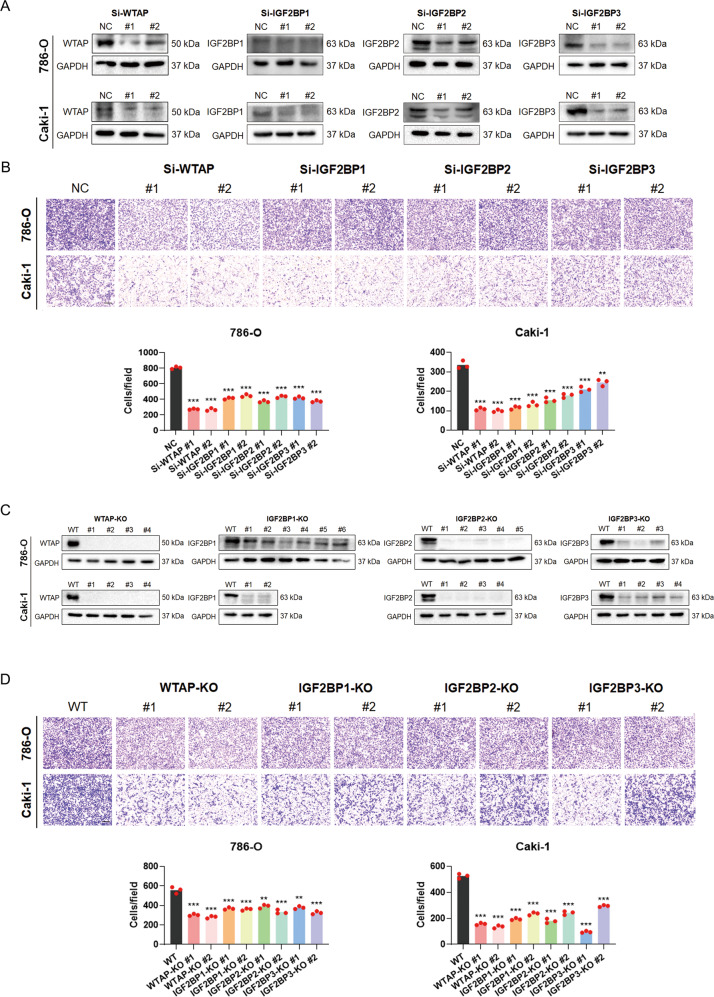
WTAP and IGF2BPs promote RCC migration in vitro. **A** The indicated genes were knocked down by siRNAs in RCC cells. **B** WTAP and IGF2BPs knockdown individually suppressed RCC migration. Scale bar 250 μm. **C** CRISPR-Cas9 mediated knockout (KO) of WTAP and IGF2BPs in RCC cells as detected by western blot. **D** WTAP and IGF2BPs KO individually suppressed RCC migration in vitro. Scale bar 250 μm. *P < 0.05; **P < 0.01; ***P < 0.001.

To further characterize the function of WTAP and IGF2BPs in RCC, siRNAs experiments were conducted to silence their gene expression and knockdown efficiency was verified using different siRNA constructs (Fig. 3A). Considering that high levels of WTAP and IGF2BPs are associated with high cancer mortality, we performed a migration assay in vitro. The results showed that knockdown of WTAP and IGF2BPs suppressed cell migration in RCC cells (Fig. 3B). To confirm these findings, we exploited the CRISPR/Cas9 technology to generate WTAP and IGF2BPs knockout (KO) 786-O and CAKI-1 cells (Fig. 3C). Consistently, WTAP KO and IGF2BPs KO cells showed decreased migration ability than control cells (Fig. 3D). Collectively, these data revealed that WTAP and IGF2BPs promote RCC metastasis.

**Fig. 4:**
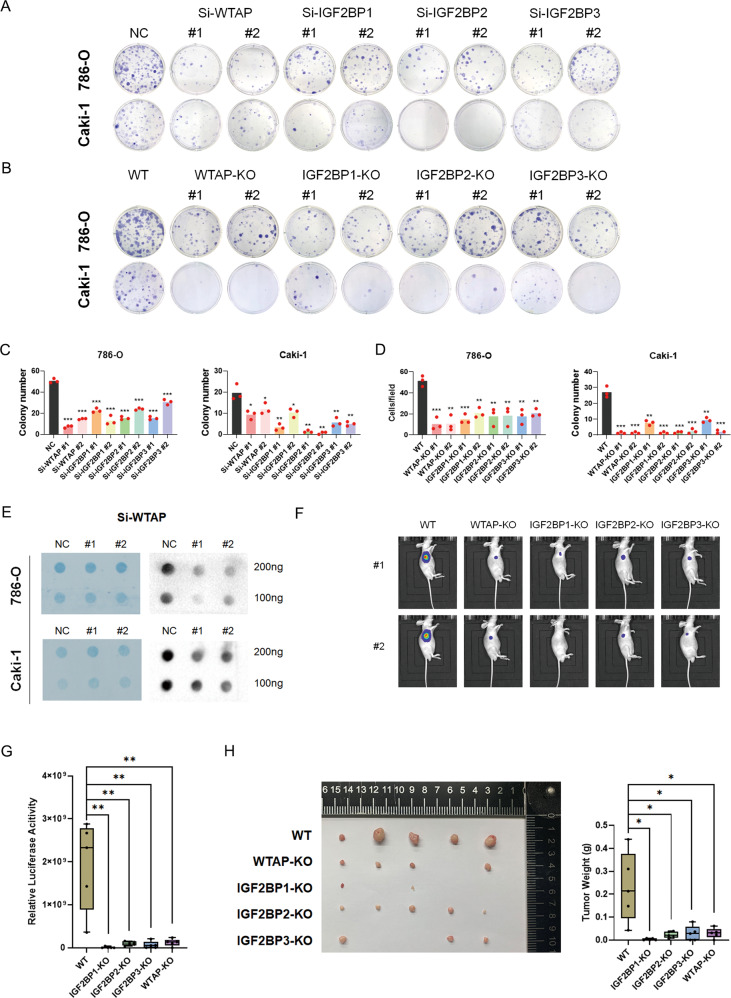
WTAP and IGF2BPs promote RCC proliferation in vitro and in vivo. **A**–**D** Either knockout or knockdown of WTAP and IGF2BPs inhibited RCC colony formation abilities. **E** M^6^A modification levels decreased in WTAP knockdown RCC cells. **F**, **G** In vivo growth of RCC subcutaneous tumor xenografts based on bioluminescence imaging. **H** Tumor weights and photographs of xenograft tumors in each group. Values in **G** and **H** are mean ± SEM. *P < 0.05; **P < 0.01; ***P < 0.001.

Consistent with the reported function of WTAP in m^6^A modification, depletion of WTAP resulted in a substantial decrease in m^6^A abundance in total RNA as detected by m^6^A dot blot assay (Fig. 4E). Knockdown or Knockout of WTAP and IGF2BPs markedly suppressed the colony-formation ability of cells (Fig. 4A–D). Notably, WTAP KO and IGF2BPs KO suppressed tumor growth in both subcutaneous and orthotopic transplantation models in immunodeficient mice (Fig. 4F–H and Supplementary Fig. 1A, B). These data demonstrated that WTAP and IGF2BPs acted as oncogenes in RCC.

### WTAP-mediated m^6^A modification maintains the enhancement of S1PR3 mRNA stability by IGF2BPs

**Fig. 5:**
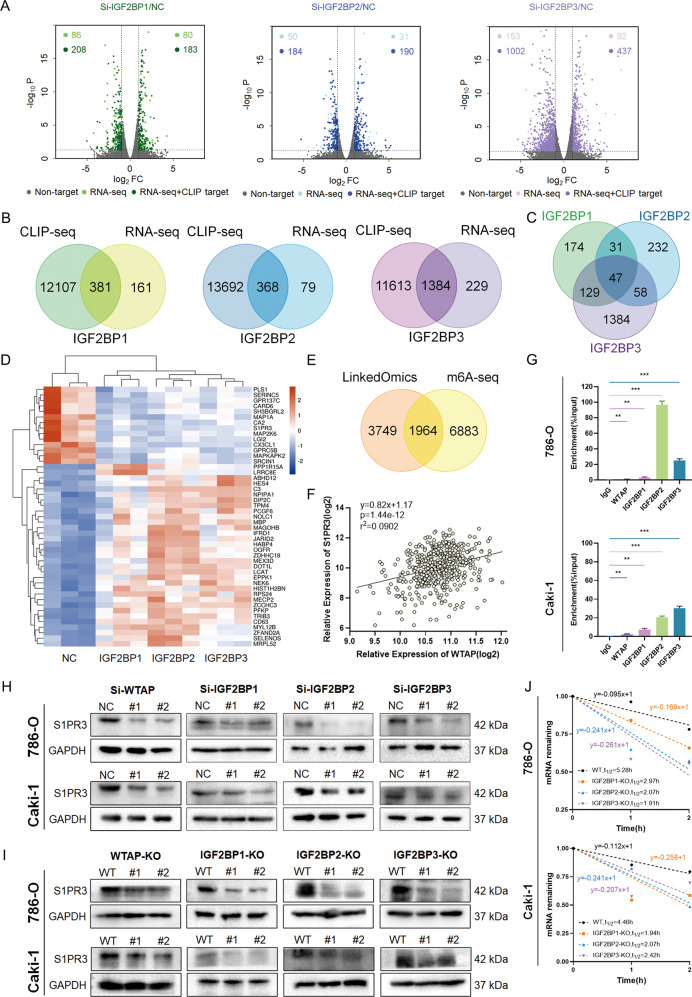
S1PR3 is the downstream target of IGF2BPs and WTAP. **A** Volcano plots displaying enrichment of significantly differentially expressed genes in IGF2BPs knockdown vs. control 786-O cells. **B** Venn diagrams showing the number of overlapping genes identified by RNA-seq and CLIP-seq. **C**, **D** Venn diagrams and heat maps showing the number of overlapping genes identified by IGF2BPs knockdown. **E** Venn diagrams showing the number of genes identified by correlation analysis and m^6^A-seq. **F** Correlation analysis of gene expression levels between WTAP and S1PR3. **G** RIP-PCR assays showing the association of WTAP and IGF2BPs with S1PR3. **H**, **I** Either knockdown or knockout of WTAP and IGF2BPs decreased protein levels of S1PR3. **J** IGF2BPs knockout reduced S1PR3 mRNA half-life in RCC cells. Values in **G** are mean ± SEM. *P < 0.05; **P < 0.01; ***P < 0.001.

To determine the downstream targets of WTAP and IGF2BPs, we performed RNA sequencing (RNA-seq) in 786-O cells with IGF2BPs knockdown and control. The results indicated that 294 transcripts were markedly downregulated following IGF2BP1 knockdown (*P* < 0.05), 234 transcripts were significantly decreased following IGF2BP2 knockdown and 1155 transcripts were decreased following IGF2BP3 knockdown (Fig. 5A and Supplementary Table S2). Among the transcripts, over 70% overlapped with reported CLIP-seq target genes (Fig. 5B) [28]. The 208, 184, and 1002 transcripts identified by RNA-seq and CLIP-seq methods were considered important targets. The three IGF2BP proteins shared 47 target genes and 14 genes were markedly decreased in IGF2BPs knockdown groups (Fig. 5C, D). Considering that WTAP was one of the crucial m^6^A writers, we searched the LinkedOmics database and found the WTAP positively correlated gene set. Among the gene set, about 34% overlapped with published m^6^A-seq genes (Fig. 5E) [29]. Integrative analysis that combined IGF2BPs high-confidence targets with m^6^A-seq data and LinkedOmics database was performed to identify potential candidate genes. S1PR3 was selected as the candidate gene while S1PR3 was markedly positively correlated with WTAP (Fig. 5F). To further confirm the correlation among WTAP, IGF2BPs, and S1PR3, RNA immunoprecipitation (RIP) assay was performed. The results indicated that WTAP and IGF2BPs directly bound to the S1PR3 mRNA (Fig. 5G). Consistently, the expression of S1PR3 was lower in WTAP KO and IGF2BPs KO cells than in control cells and knockdown of individual WTAP or IGF2BPs (Fig. 5H, I). Since IGF2BP proteins can regulate gene expression at the mRNA and translation levels, we sought to determine the effect of IGF2BPs on S1PR3 mRNA stability. Results showed that the knockout of IGF2BPs in RCC cells markedly decreased the half-life of S1PR3 mRNA (Fig. 5J).

**Fig. 6:**
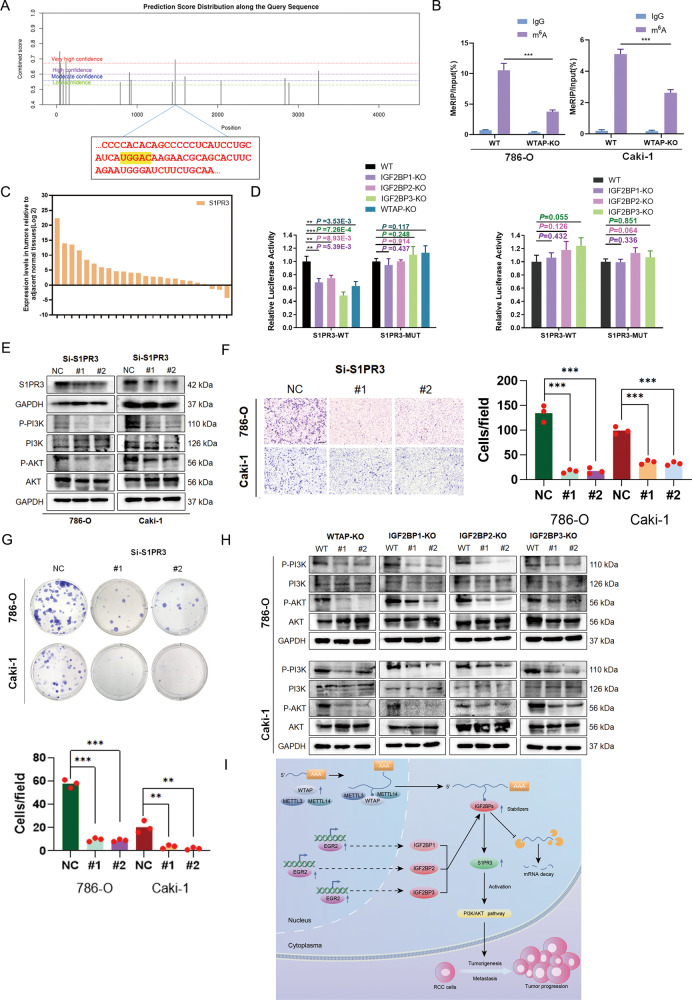
WTAP-mediated m^6^A modification of S1PR3 maintains the IGF2BPs-induced regulation of RCC proliferation and metastasis. **A** The predicted sites in S1PR3 mRNA in the SRAMP database. **B** Enrichment of m^6^A modification on S1PR3 as detected by MeRIP-PCR assay. **C** S1PR3 were elevated in 21 (87.5%) RCC tissues relative to adjacent nonmalignant tissues. **D** Relative luciferase activities of S1PR3-WT of S1PR3-MUT in WTAP KO 786-O cells with IGF2BPs knockdown. **E** The protein levels of S1PR3 and PI3K/AKT pathway following S1PR3 knockdown. **F**, **G** Knockdown of S1PR3 markedly suppressed migration and proliferation viabilities of RCC cells. Scale bar 250 μm. **H** Knockout of WTAP and IGF2BPs regulate PI3K/AKT pathway. **I** Schematic diagram of IGF2BP-mediated regulation of m^6^A-modified S1PR3 mRNA. Values in **B** and **D** are mean ± SEM. *P < 0.05; **P < 0.01; ***P < 0.001.

We further determined whether IGF2BP-mediated S1PR3 regulation is m^6^A-dependent. As reported previously, IGF2BP proteins preferentially bind to the “UGGAC” consensus sequence and the bind sites are highly enriched near stop codons and in the 3’ UTRs [14]. Thus, we exploited SRAMP prediction tool to predict the potential bind sites of S1PR3. Interestingly, one of the putative binding sites contained “UGGAC” consensus sequence and located near stop codons (Fig. 6A). Methylation RNA immunoprecipitation (MeRIP) assay was performed with negative control IgG and m^6^A antibodies, and RT-PCR was performed using the primers specific for the predicted S1PR3-binding site. As expected, we observed a decrease in m^6^A methylation level of S1PR3 in WTAP KO cells which confirmed that m^6^A modification of S1PR3 is catalyzed by the predominant catalytic subunit WTAP (Fig. 6B). Especially, S1PR3 was highly expressed in 21 (87.5%) clinical samples (Fig. 6C). We next inserted the 44-nt wild-type or mutant sequence containing the binding site into a dual-luciferase reporter (Supplementary Fig. 3E). The dual-luciferase reporter assay showed decreased luciferase activity following individual IGF2BPs knockout in wild-type reporter, and such decrease was almost completely abrogated by mutation in the m^6^A consensus sites (Fig. 6D). Consistently, WTAP knockout, similar to individual IGF2BPs knockout, inhibited firefly luciferase activity. In addition, IGF2BP knockdown-mediated decrease of luciferase activity was completely blocked by WTAP knockout (Fig. 6D). Taken together, these results revealed that WTAP-mediated m^6^A modification maintained the enhancement of S1PR3 stability by IGF2BP proteins.

### S1PR3 is responsible for the IGF2BPs-induced regulation of RCC proliferation and metastasis

To determine whether IGF2BPs-induced regulation of cell proliferation and migration in RCC relies on S1PR3, the role of S1PR3 on the proliferation and metastasis of RCC cells by knocking down its expression by siRNAs. The efficiency of siRNAs silencing is shown in Fig. 6E. We found that S1PR3 knockdown markedly inhibited proliferation and migration abilities of 786-O and CAKI-1 cells in vitro (Fig. 6F, G). S1PR3 has been reported to regulate PI3K/AKT pathway and our results consistently indicated that downregulation of S1PR3 regulated the PI3K/AKT signaling pathway (Fig. 6E). Notably, knockdown or knockout of IGF2BPs or WTAP regulated the PI3K/AKT signaling pathway (Fig. 6H and Supplementary Fig. 2C). Similar results were observed with EGR2 knockdown (Supplementary Fig. 3D). In contrast, knockdown of EGR2 had no effect on WTAP expression (Supplementary Fig. 3D). To test the hypothesis that IGF2BPs-induced regulation of RCC proliferation and migration is relevant for S1PR3 downregulation, we performed rescue experiments by overexpressing S1PR3 in WTAP KO and IGF2BPs KO cells. Results showed that forced expression of S1PR3 partly abrogated the inhibitory effect WTAP KO and IGF2BP KO on colony-formation rates (Supplementary Fig. 2B). Similar results were obtained in transwell experiments in which S1PR3 was used to rescue WTAP KO and IGF2BPs KO (Supplementary Fig. 2A). The protein expression levels of S1PR3 in the above rescue experiments are shown in Supplementary Fig. 1C. In summary, WTAP and IGF2BP proteins promote tumorigenesis and metastasis by enhancing the stability of S1PR3 and regulating S1PR3-PI3K/AKT pathway (Fig. 6I).

## Discussion

Prior studies have noted the importance of m^6^A modification in multiple cellular processes and pathogenesis of various diseases [30]. We previously found that METTL3 promotes the carcinogenesis and metastasis of prostate cancer and bladder cancer [24, 25]. The incidence of renal cell cancer has been rising over the years. How m^6^A regulates the pathomechanisms of renal cancer remains obscure. Here, we studied the role of WTAP and IGF2BP proteins in RCC using specimens from 24 patients with RCC. We found four m^6^A regulators that were mostly upregulated, although IGF2BP1 was either minimally or not detected in some tissues. In addition, high expression of WTAP and IGF2BPs correlated with the poor prognosis of RCC patients. Functionally, WTAP and IGF2BPsl promoted the migration in vitro and growth of RCC cells in vitro and in vivo. Mechanistically, WTAP regulated S1PR3 in a m^6^A-mediated and IGF2BPs-associated manner. Furthermore, WTAP/m^6^A/IGF2BPs/S1PR3 regulated renal cancer proliferation and metastasis in a PI3K/AKT-dependent pattern.

WTAP is a known oncogene in various tumors [31, 32]. WTAP enhanced mRNA stability of CDK2 in RCC [33], but the role of WTAP as an m^6^A writer in RCC has not been explored. The m^6^A dot blot experiment indicated that WTAP regulates m^6^A modification since the overall of the m^6^A level was markedly declined by WTAP knockdown. Next, we explored the downstream targets of WTAP-mediated m^6^A modification based on data from LinkedOmics, RNA-seq, and m^6^A-seq. Results showed that S1PR3 was a target of WTAP. Subsequently, S1PR3 was modified in the 3’UTRs via WTAP-mediated m^6^A methylation as determined by dual-luciferase reporter assay and MeRIP-qPCR. In addition, our findings clarified that WTAP could promote RCC malignant development through regulating S1PR3 expression via motivating PI3K/AKT pathway. All these data implied the effects of WTAP on RCC carcinogenesis depended on different downstream molecules.

IGF2BP proteins have been recognized as a new family of m^6^A readers, regulate multiple biological processes and cancers [34–36]. However, its role as an m^6^A reader remains unknown. Herein, we demonstrate that IGF2BPs is upregulated in RCC tissues due to the transcription factor EGR2, which directly bound to the promoter region of IGF2BPs. RIP assays confirmed the direct binding of IGF2BPs to the S1PR3 mRNA, and the stability of S1PR3 was reduced when IGF2BPs were individually silenced. The direct effects of IGF2BPs on S1PR3 mRNA were abrogated by WTAP silencing as revealed by western blot and dual-luciferase reporter assay. Thus, IGF2BP proteins enhanced the S1PR3 mRNA stability in a m^6^A-dependent manner.

As a member of the EDG family of receptors, S1PR3 regulates angiogenesis and vascular endothelial cell function [37]. It has also been shown to function as an oncogene in various cancers. For example, S1PR3 was elevated in osteosarcoma and inhibited the phosphorylation of YAP, and promoted the nuclear translocation of YAP [38]. Moreover, S1PR3 can be stimulated by S1P to activate the Notch pathway in a Notch ligand-independent manner [39]. In addition, PI3K/AKT signaling has been targeted in cancer therapeutics, and previous reports show that S1PR3 regulates the PI3K/AKT pathway [40, 41]. Consistently, our results indicated that S1PR3 activated PI3K phosphorylation and enhanced cancer initiation and progression. However, as a downstream of WTAP/IGF2BPs, forced expression of S1PR3 only partly abrogated the inhibition of colony rates and migration in WTAP KO and IGF2BPs KO cells, implying that there are other molecular mechanisms of WTAP/IGF2BPs m^6^A axis in RCC.

## Conclusions

In summary, WTAP and IGF2BPs are overexpressed in renal cancer, which correlates with a worse prognosis of RCC patients. WTAP and IGF2BPs serve as potential prognostic factors for patients with RCC. As a transcription factor, EGR2 maintains high expression of IGF2BP proteins in RCC cells. WTAP and IGF2BPs decreased the cell proliferation and migration of RCC cells via regulating the stability of S1PR3 mRNA in a m^6^A-dependent manner. S1PR3 regulated RCC initiation and progression by regulating the PI3K/AKT pathway. Altogether, these results demonstrated the oncogenic role of WTAP/m^6^A/IGF2BPs/S1PR3 in RCC.

## Supplementary information

Supplementary Fig.1

Supplementary Fig.2

Supplementary Fig.3

Supplementary Table S1

Supplementary Table S2

Supplementary Table S3

## Data Availability

All data generated or analyzed during this study are included in this published article and its supplementary information files.
