# Supplementary figures and images for: EGR2-mediated regulation of m6A reader IGF2BP proteins drive RCC tumorigenesis and metastasis via enhancing S1PR3 mRNA stabilization
Source: Cell Death Dis. 2021 Jul 29;12(8):750. doi: 10.1038/s41419-021-04038-3 (PMC8322060; doi:10.1038/s41419-021-04038-3)

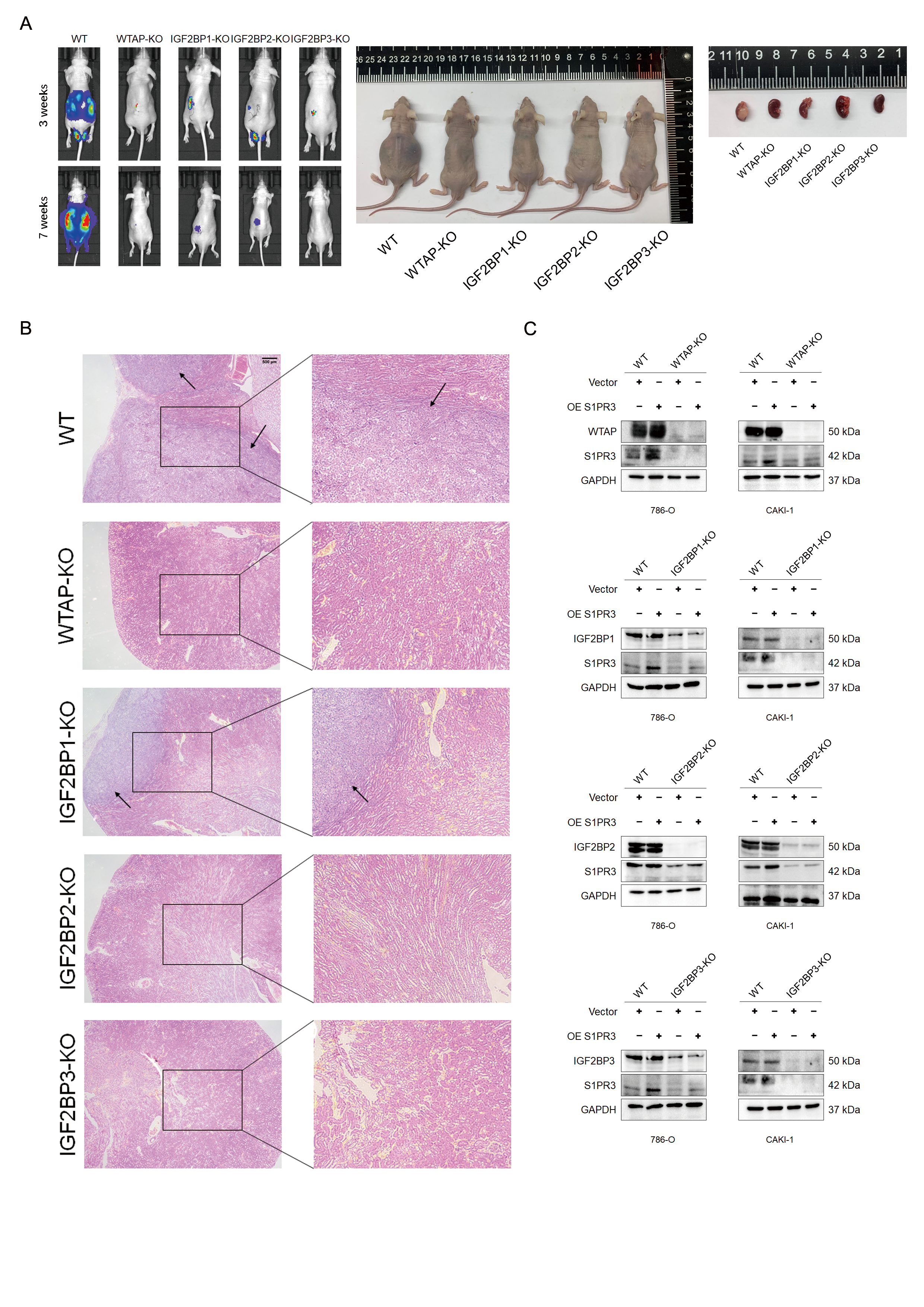

Supplement: Supplementary file 1 — Supplementary Fig.1 [file 41419_2021_4038_MOESM1_ESM.png]

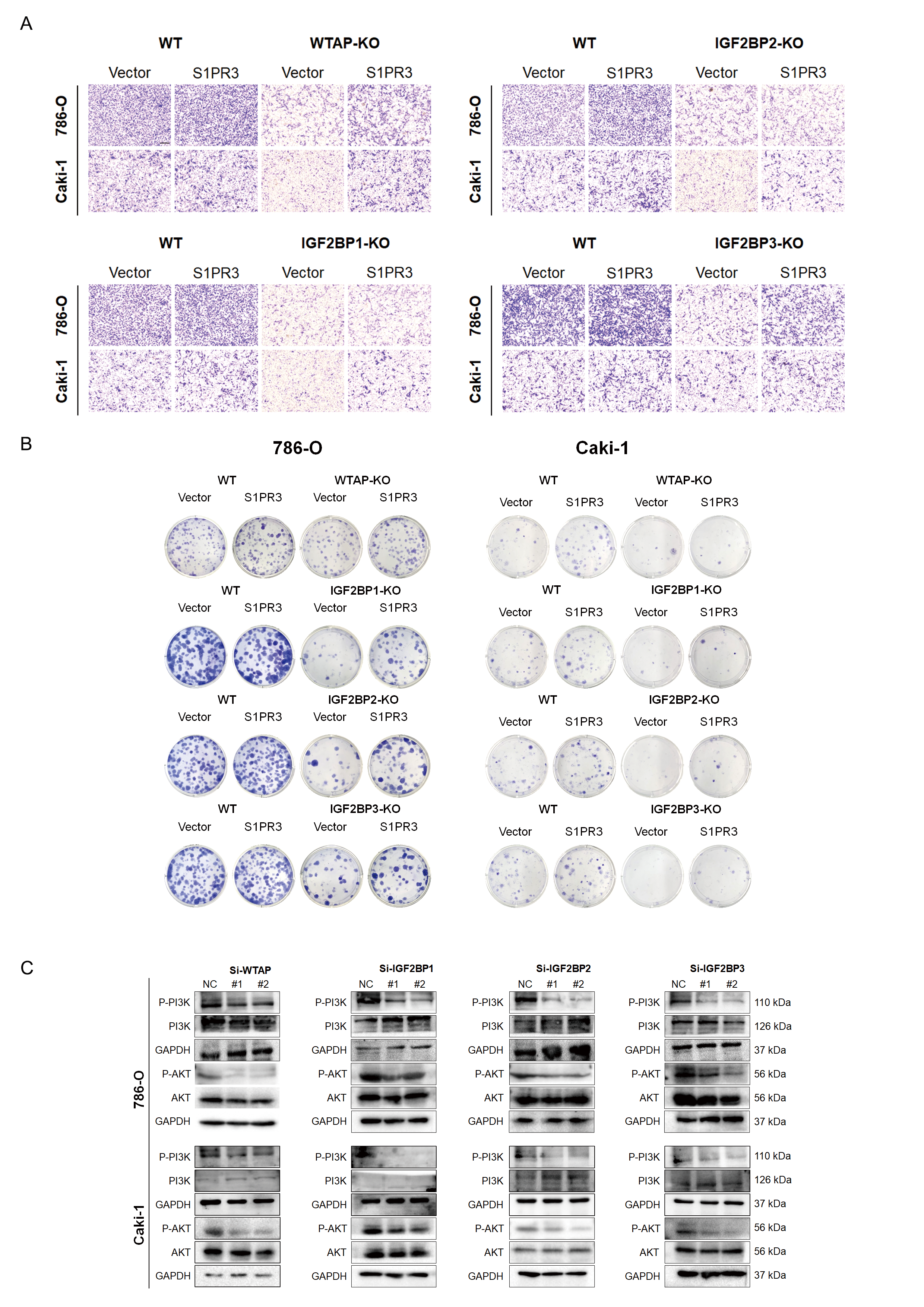

Supplement: Supplementary file 2 — Supplementary Fig.2 [file 41419_2021_4038_MOESM2_ESM.png]

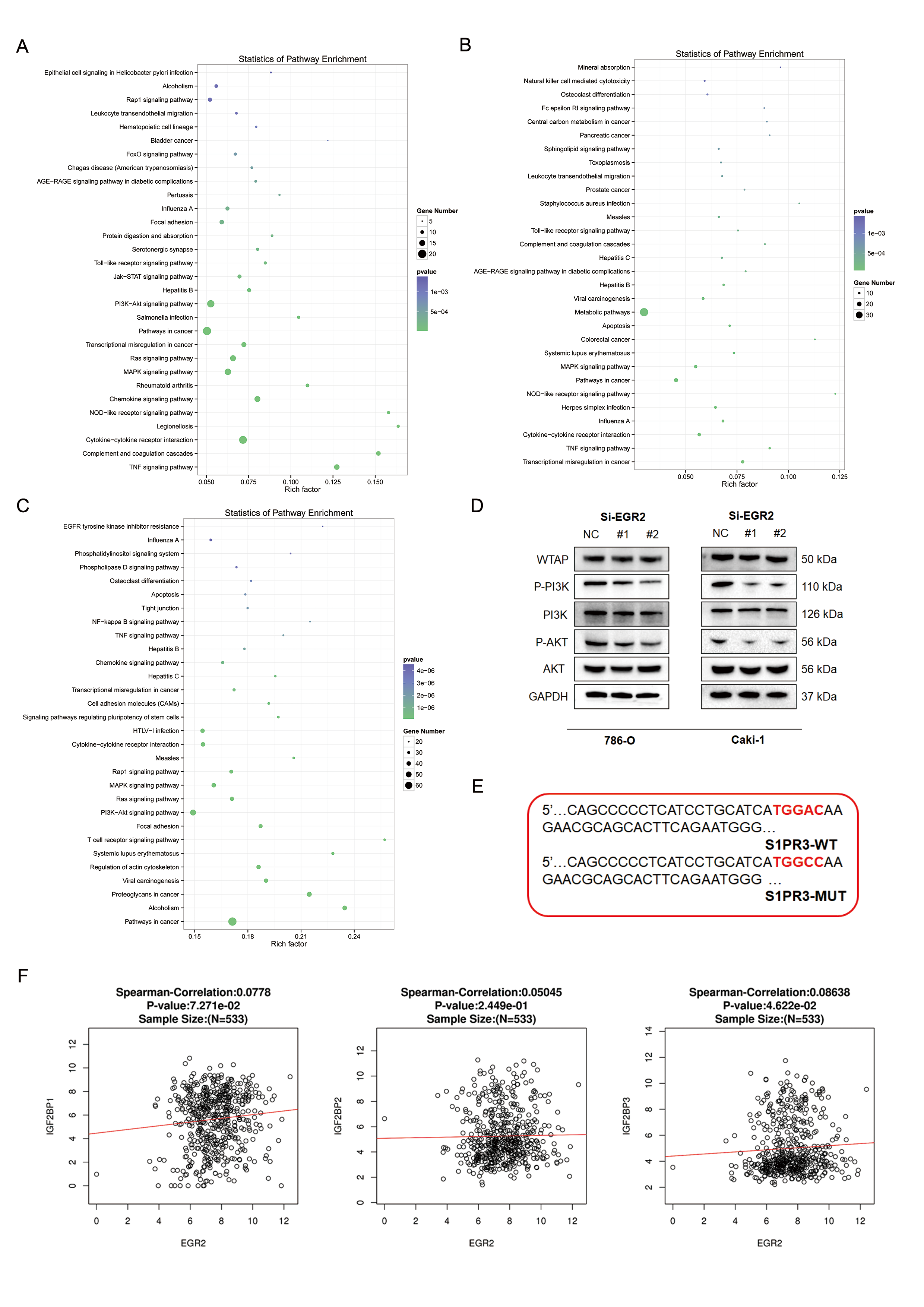

Supplement: Supplementary file 3 — Supplementary Fig.3 [file 41419_2021_4038_MOESM3_ESM.png]
