# Supplementary material for: EGR2-mediated regulation of m6A reader IGF2BP proteins drive RCC tumorigenesis and metastasis via enhancing S1PR3 mRNA stabilization
Source: Cell Death Dis. 2021 Jul 29;12(8):750. doi: 10.1038/s41419-021-04038-3 (PMC8322060; doi:10.1038/s41419-021-04038-3)
Supplement: Supplementary file 4 — Supplementary Table S1 [file 41419_2021_4038_MOESM4_ESM.docx]

**Supplemental Table S1 The oligonucleotides used in this study.**

| **Name^a^** | **Sequence (5’->3’) ^b^** |
| --- | --- |
| NC (sense) | ACUACUGAGUGACAGUAGA |
| Si-WTAP (sense) | GCGAAGUGUCGAAUGCUUATT |
|  | GGGUAUGCAGAGUACCAUUTT |
| Si-IGF2BP1(sense) | GGCCCAUAAUAACUUUGUATT |
|  | GCUCCCUAUAGCUCCUUUATT |
| Si-IGF2BP2(sense) | CAGUUUGAGAACUACUCCUTT |
|  | GAAACAGGGACCAAGAUAATT |
| Si-IGF2BP3(sense) | GGAUUCGGAAACUUCAGAUTT |
|  | CACCUUGAAAGUAGCCUAUTT |
| Si-S1PR3 (sense) | CCGUGCUCUUCUUGGUCAUTT |
|  | GCAUCGCUUACAAGGUCAATT |
| Sg-WTAP | CGAAGAACCTCTTCCCAAGA |
|  | CAAGAGATGAGTTAATTCTA |
|  | TGAAGCATATGTACAAGCTT |
| Sg-IGF2BP1 | ATATTCCACCCCAGCTCCGA |
|  | ACGCTTAGAGATTGAACATT |
|  | TGAATGTCACCTATTCCAAC |
| Sg-IGF2BP2 | ATGCCCGCTTAGCTTCTCCA |
|  | ACATCCCTCCTCACCTGCAG |
|  | CGATGATGGCACCAACAAAC |
| Sg-IGF2BP3 | ATATCCCGCCTCATTTACAG |
|  | GGCCATTTCATCAGGGATAT |
|  | TGATTTGCCTCTGCGCCTGC |
| IGF2BP1-F | GCGGCCAGTTCTTGGTCAA |
| IGF2BP2-F | AGTGGAATTGCATGGGAAAATCA |
| IGF2BP3-F | TATATCGGAAACCTCAGCGAGA |
| IGF2BP1-R | TTGGGCACCGAATGTTCAATC |
| IGF2BP2-R | CAACGGCGGTTTCTGTGTC |
| IGF2BP3-R | GGACCGAGTGCTCAACTTCT |
| WTAP-F | CTTCCCAAGAAGGTTCGATTGA |
| WTAP-R | TCAGACTCTCTTAGGCCAGTTAC |
| S1PR3-F | CGGCATCGCTTACAAGGTCAA |
| S1PR3-R | GCCACGAACATACTGCCCT |
| Me-S1PR3-F | AAGGTCAAGGAAGACCTGCCC |
| Me-S1PR3-R | AAGATCCCATTCTGAAGTGCTGC |
| pmirGLO primer | ACACGGTAAAACCATGAC |
| S1PR3-WT-F | cCCCCCTCATCCTGCATCATGGACAAGAACGCAG  CACTTCAGAATg |
| S1PR3-WT-R | tcgacATTCTGAAGTGCTGCGTTCTTGTCCATGATG |
|  | CAGGATGAGGGGGgagct |
| S1PR3-MUT-F | cCCCCCTCATCCTGCATCATGGCCAAGAACGCAG |
|  | CACTTCAGAATg |
| S1PR3-MUT-R | tcgacATTCTGAAGTGCTGCGTTCTTGGCCATGATG |
|  | CAGGATGAGGGGGgagct |
| IGF2BP1-WT-F | ctagcGAGCAGCCCCCTCCCCCACCGCCCAGACGGGGTGCGACCGCCCACGTGTCGCCCCTTGCCCAGTCGGGTCCTTCCCTCGGGCTCCGGGAGCCGGAGGATCCGGAATGAGTa |
| IGF2BP1-WT-R | agcttACTCATTCCGGATCCTCCGGCTCCCGGAGCCCGAGGGAAGGACCCGACTGGGCAAGGGGCGACACGTGGGCGGTCGCACCCCGTCTGGGCGGTGGGGGAGGGGGCTGCTCg |
| IGF2BP2-WT-F | ctagcCTCTCCGCGCTCACTCGCAGCCCCTCTCCCTCCCACGCCCGCGGCTCCCCGTCGCCCCCTCGCGCGCTCCCACCCGCGCCCGGGGCGCGCCTTCCCCGCCCCGCCGCTCGa |
| IGF2BP2-WT-R | agcttCGAGCGGCGGGGCGGGGAAGGCGCGCCCCGGGCGCGGGTGGGAGCGCGCGAGGGGGCGACGGGGAGCCGCGGGCGTGGGAGGGAGAGGGGCTGCGAGTGAGCGCGGAGAGg |
| IGF2BP3-WT-F | ctagcCCACCGCGAGCGCAGGCGGAGGCGGAGGAGGTGTCGCCCAGACACCCGCCCAGGACGCTGCGGGATCCCATCCCCCACCACTCCGGGGCCGGGCCGCCCCCACTCGCGCGa |
| IGF2BP3-WT-R | agcttCGCGCGAGTGGGGGCGGCCCGGCCCCGGAGTGGTGGGGGATGGGATCCCGCAGCGTCCTGGGCGGGTGTCTGGGCGACACCTCCTCCGCCTCCGCCTGCGCTCGCGGTGGg |
| IGF2BP1-MUT-F | ctagcGAGCAGCCCCCTCCCCCACCGCCCAGACGGGGTGCGATCGCCCCTTGCCCAGTCGGGTCCTTCCCTCGGGCTCCGGGAGCCGGAGGATCCGGAATGAGTa |
| IGF2BP1-MUT-R | agcttACTCATTCCGGATCCTCCGGCTCCCGGAGCCCGAGGGAAGGACCCGACTGGGCAAGGGGCGATCGCACCCCGTCTGGGCGGTGGGGGAGGGGGCTGCTCg |
| IGF2BP2-MUT-F | ctagcCTCTCCGCGCTCACTCGCAGCCCCTCTCCGCGGCTCCCCGTCGCCCCCTCGCGCGCTCCCACCCGCGCCCGGGGCGCGCCTTCCCCGCCCCGCCGCTCGa |
| IGF2BP2-MUT-R | agcttCGAGCGGCGGGGCGGGGAAGGCGCGCCCCGGGCGCGGGTGGGAGCGCGCGAGGGGGCGACGGGGAGCCGCGGAGAGGGGCTGCGAGTGAGCGCGGAGAGg |
| IGF2BP3-MUT-F | ctagcCCACCGCGAGCGCAAGGAGGTGTCGCCCAGACACCCGCCCAGGACGCTGCGGGATCCCATCCCCCACCACTCCGGGGCCGGGCCGCCCCCACTCGCGCGa |
| IGF2BP3-MUT-R | agcttCGCGCGAGTGGGGGCGGCCCGGCCCCGGAGTGGTGGGGGATGGGATCCCGCAGCGTCCTGGGCGGGTGTCTGGGCGACACCTCCTTGCGCTCGCGGTGGg |

^a^ F, forward primer; R, reverse primer.

^b^ Restriction sites are in bold
